# Studies on Surfactants, Cosurfactants, and Oils for Prospective Use in Formulation of Ketorolac Tromethamine Ophthalmic Nanoemulsions

**DOI:** 10.3390/pharmaceutics13040467

**Published:** 2021-03-30

**Authors:** Shahla S. Smail, Mowafaq M. Ghareeb, Huner K. Omer, Ali A. Al-Kinani, Raid G. Alany

**Affiliations:** 1 Drug Discovery, Delivery and Patient Care (DDDPC) Theme, Department of Pharmacy, Kingston University, Kingston upon Thames, London KT1 2EE, UK; R.Alany@kingston.ac.uk; 2 Department of Pharmaceutics, College of Pharmacy, Hawler Medical University, Kurdistan Region, Erbil 44001, Iraq; huner.omer@hmu.edu.krd; 3 Department of Pharmaceutics, College of Pharmacy, University of Baghdad, Baghdad 10011, Iraq; Mowafaq.abd@copharm.uobaghdad.edu.iq; 4 School of Pharmacy, The University of Auckland, Auckland 1023, New Zealand

**Keywords:** nanoemulsion, ketorolac tromethamine, physicochemical properties, solubility, HET-CAM assay

## Abstract

Nanoemulsions (NE) are isotropic, dispersions of oil, water, surfactant(s) and cosurfactant(s). A range of components (11 surfactants, nine cosurfactants, and five oils) were investigated as potential excipients for preparation of ketorolac tromethamine (KT) ocular nanoemulsion. Diol cosurfactants were investigated for the effect of their carbon chain length and dielectric constant (DEC), Log *P*, and HLB on saturation solubility of KT. Hen’s Egg Test—ChorioAllantoic Membrane (HET-CAM) assay was used to evaluate conjunctival irritation of selected excipients. Of the investigated surfactants, Tween 60 achieved the highest KT solubility (9.89 ± 0.17 mg/mL), followed by Cremophor RH 40 (9.00 ± 0.21 mg/mL); amongst cosurfactants of interest ethylene glycol yielded the highest KT solubility (36.84 ± 0.40 mg/mL), followed by propylene glycol (26.23 ± 0.82 mg/mL). The solubility of KT in cosurfactants was affected by four molecular descriptors: carbon chain length, DEC, log *P* and HLB. KT solubility was directly proportional to DEC and the HLB yet, inversely proportional to carbon chain length and log *P*. All surfactants, except Labrasol ALF, were non-irritant. The majority of cosurfactants were slightly irritant, butylene glycol was a moderate irritant, pentylene and hexylene glycols were strong irritants. These findings will inform experiments aimed at developing NE formulations for ocular administration of KT.

## 1. Introduction

Nanoemulsions (NE) are optically isotropic, colloidal dispersions with droplet sizes ranging from 20 to 500 nm [1,2], mainly consisting of oil, surfactants (surfactants and cosurfactants), and water [3,4]. They are characterized by a transparent/translucent appearance and long-term stability which is mainly due to their extremely small droplet size [5,6]. NE systems have attracted increasing interest as potential carriers for drugs and biologics. They are suitable carriers for both hydrophilic, lipophilic and amphiphilic drugs; may control the release and protect susceptible compounds like antioxidants, vitamins and shield them from external degradation triggers, such as light, pH, and temperature [7,8].

NEs are promising for ocular delivery of drugs, because of their ability to sustain ocular drug release, which in turn improves ocular bioavailability, increasing corneal absorption and drug permeation through membranes of the eye, and reducing the frequency of drug administration, thus promoting better patient compliance [4,9]. The ocular bioavailability of topically applied drugs is usually from (0.1–5%) mainly due to loss via nasolacrimal drainage as well poor corneal permeability [10,11]. NEs have attracted increasing interest as a vehicle for topical ocular drug delivery through extending drug release, prolonging precorneal retention, and increasing corneal permeability. Cationic NEs of rifampicin with prolonged residence time on the eye have been prepared to combat ocular tuberculosis [12], ibuprofen-loaded sustained-release NEs have been developed to act on inflammatory components of dry eye disease [13]. NEs have been used as a biocompatible carrier for ocular delivery of travoprost with improved pharmacodynamic and pharmacokinetic properties [14]; tacrolimus cationic NEs provided prolonged retention time which is desirable in ocular inflammatory diseases [2]; moxifloxacin NE has been prepared with a significant increase of bioavailability, reduced dosing frequency, and improved therapeutic activity [15]; anti-glaucoma non-irritant NEs of brinzolamide have been prepared with enhanced corneal permeability [16].

Surfactants and cosurfactants are essential components of NE systems. The surfactant is adsorbed at the oil/water interface causing decreased surface tension, leading to droplet size reduction, and NE formation [17]. The combination of surfactant and cosurfactant used in the preparation of nanoemulsions is essential for producing the ultralow interfacial tension needed to reduce droplet size to the nanometer range [5,18]. Using the lowest possible concentrations of surfactants and cosurfactants in the preparation of NEs is essential to reduce the possibility of conjunctival irritation and toxicity to the cornea, and ocular surface [19]. On most occasions, the use of surfactants alone is insufficient to reduce the interfacial tension to levels required for NE formation, so a cosurfactant is used [20].

Cosurfactants have unique molecular characteristics; a short to medium hydrophobic chain, weak amphiphilic nature, and terminal small hydrophilic group (such as hydroxyl group characteristic of low molecular weight alcohols and diols). These properties increase the tendency of cosurfactants to interact with surfactant monolayers at interfaces, perturbing the packing and long-range order of surfactant molecules and promoting better interface fluidity and curvature in favor of NE formation. Furthermore, the balanced amphiphile nature of these molecules promotes their selective partitioning between phases, changing their composition and thereby their relative hydro/lipophilicity of the immiscible phases to improve better miscibility [21].

Non-ionic surfactants are more suited for ocular preparations due to their lower ocular toxicity and better tolerability; the tendency of surfactants to cause ocular irritation is as follows: non-ionic < Zwitterionic < anionic < cationic [22]. Furrer et al. reported similar findings when they investigated eight different surfactants for ocular irritation in both rabbit and mice models [23].

The physicochemical properties of surfactants and cosurfactants such as dielectric constant (DEC), the logarithm of the octanol/water partition coefficient (log *P*), molecular weight, carbon-chain length, and branching and carbon content weight percent (C%) may impact the formation of NE and their ability to solubilize drugs. The hydrophile-lipophile balance value (HLB) is regarded as an empirical guide for the selection of a good surfactant and cosurfactant suitable for NE preparation [20,24,25]. The percentage weight of hydrophilic and lipophilic moieties of a surfactant molecule is important for determining the behavior of the surfactant [26]. The higher the HLB of a surfactant, the higher its aqueous solubility [22]. Log *P* is a measure of lipophilicity [27], a linear relationship has been found between the lipophilicity as a function of log *P* and the tendency for solubilization [25].

**Figure 1:**
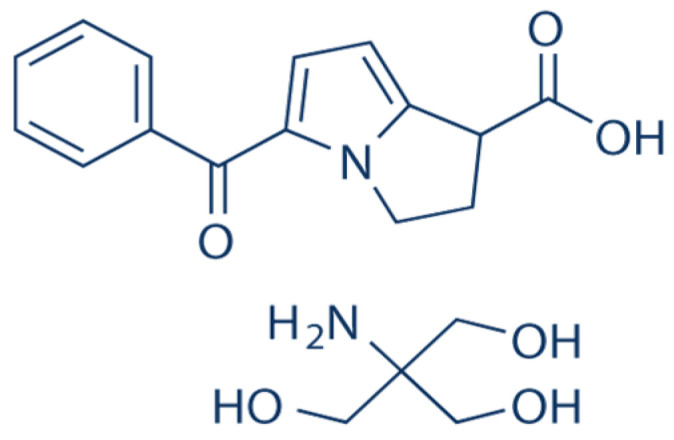
Ketorolac tromethamine (KT) chemical structure.

Ketorolac tromethamine (KT) (Figure 1) is a first-generation, non-steroidal anti-inflammatory drug with potent analgesic activity; it exists in three crystalline forms with equal aqueous solubility. It acts by non-specific inhibition of prostaglandin synthesis through the competitive blocking of cyclooxygenase (COX) enzyme. Ketorolac is effective in management of postoperative inflammation and seasonal allergic conjunctivitis, without affecting herpes simplex, fungal and bacterial infection of the eye [28,29]. It is commercially available as a sterile aqueous solution (0.4–0.5% *w*/*v*) for use as eye drops, where it is applied topically up to four times a day. The need for multiple application of KT eye drops along with their undesirable side effects of severe burning sensation, stinging and itching of the eye results in poor patient adherence, often leading to failure of pain and inflammation management after cataract or corneal refractive surgery [30].

Many attempts have been made to develop KT formulations with improved efficacy. Poloxamer based in situ gels with sustained release properties and enhanced bioavailability [31]; chitosan-based nanoparticles with increased ocular retention time and enhanced permeability [32]; and poloxamer 407-based nanocellulose grafted collagen (CGC) to sustained KT release are some examples [30]. However, none of the aforementioned approaches addressed the patient’s discomfort associated with the topical ocular application of KT eye drops, nor did they improve patient compliance. KT NE could serve to prolong ocular residence time, which would enable the reduction of the required dose [13]. The KT dose reduction along with the emollient/lubricant characteristics of the proposed NE formulation is likely to help minimize the burning and stinging associated with conventional KT eye drops which would hopefully improve patient acceptability [33].

This manuscript reports on studies aimed at screening a range of non-ionic surfactants, cosurfactants, and oils with emphasis on their suitability for use in developing NE formulation for topical ocular administration of KT.

## 2. Materials and Methods

### 2.1. Materials

Ketorolac tromethamine (KT) was obtained from TCI chemicals (Tokyo, Japan). Fertilized white horn eggs were purchased from Heath Farm (Fakenham, UK). Transcutol P, Labrafac PG, Labrafac lipophile WL 1349, Labrafil M 1944 CS, Labrafil M 2125 CS, Labrafil M 2130 CS, and Labrasol ALF were received as a gift from Gattefosse SAS (Leon, France). Tween 20, Tween 60, Tween 80, Span 20, triacetin, ethyl oleate, dimethyl isosorbide, ethylene glycol, propylene glycol and isopropyl myristate were obtained from ACROS organics (Fair Lawn, NJ, USA). Cremophor RH 40, Cremophor EL, butylene glycol, pentylene glycol, hexylene glycol, Span 80, 2-butyl-2-ethyl-1,3-propanediol, (2,3-butanediol), NaOH were purchased From Sigma-Aldrich (Taufkirchen, Germany). Sodium chloride, sodium bicarbonate, and calcium chloride dehydrate were from Fisher Scientific UK Ltd. (Loughborough, UK). Other chemicals used were of analytical grade.

### 2.2. Methods

#### 2.2.1. Identification and Selection of Potential Surfactants and Cosurfactants Based on HLB Calculations

The choice of suitable excipients is essential in the development of NE. For this study, the selection of excipients was based on reviewing published literature, and all excipients were checked for suitability of use in ophthalmic preparations. The HLB system can be used as a systematic method for narrowing down candidates and a selection of potential surfactants and cosurfactants for preparation of a stable NE [34].

The HLB value for all surfactants and cosurfactants was determined by the Davies method [35], in which the HLB value is directly calculated from the chemical formula of the molecule (Equation (1)), and the method specifies the group number for each constituent of the molecule, the calculated values are then compared with reported values.

HLB = Σ (hydrophilic group numbers) − *n* (group number per CH_2_ group) + 7 (1)

in which, *n* is the number of -CH_2_- groups in the surfactant molecule.

Nonionic surfactants are often used for NE preparations because; they are less affected by changes in pH and ionic strength, they possess a high degree of compatibility with other components, they have a good physicochemical stability, and low toxicity [36]. In this study, a group of non-ionic surfactants were selected (Tween 20, Tween 60, Tween 80, Span 20, Span 80, Cremophor RH 40, Cremophor EL, Labrasol ALF, Labrafil M 2125 CS, Labrafil M 1944 CS, and Labrafil M 2130 CS) with different HLB values. Cosurfactants included (ethylene glycol, propylene glycol, butylene glycol, pentylene glycol, hexylene glycol, dimethyl isosorbide, 2-butyl-2-ethyl-1,3-propanediol, (2,3-butanediol), and Transcutol P), while triacetin, ethyl oleate, Labrafac PG, Labrafac lipophile WL 1349, and isopropyl myristate were used as oils.

#### 2.2.2. Determination of Selected Properties and Molecular Descriptors of Cosurfactants

The physicochemical properties of selected cosurfactants (diols) with different carbon chain lengths were studied depending on six descriptors [carbon chain length, logarithm of partition coefficient in octanol/water (log *P*), dielectric constant (DEC), molecular weight (Mwt) and carbon content weight percent (C%).

#### 2.2.3. Saturation Solubility Studies of Ketorolac Tromethamine (KT) in Surfactants, Cosurfactants, and Oils

The saturation solubility of ketorolac tromethamine (KT) was determined by adding an excess amount of drug powder to 5 mL of each oil, surfactant, cosurfactant, deionized water, and simulated tear fluid (STF), all of which were kept in tightly closed small glass tubes. Then these tubes were placed in a shaking water bath at 25 ± 0.5 °C for 72 h. After this time, the samples were filtered by using a syringe filter membrane (SLGP033RS Millipore Millex-GP Syringe Filter Unit, 0.22 µm, polyether sulfone (PES)). Once the samples had been filtered, the solubility was determined at the λ max of KT (322 nm) using a UV-visible spectrophotometer (Jenway 7315 Spectrophotometer, Bibby Scientific Ltd., Stone, UK). The filter was subjected to multiple washes followed by analysis to quantify any traces of KT being adsorbed on the filter membrane. No evidence of any KT adsorption to the filter membrane was found. The STF was made using 0.67 g sodium chloride, 0.20 g sodium bicarbonate, and 0.008 g of calcium chloride dehydrate to 100 mL distilled water q.s. (pH 7.4) [37].

#### 2.2.4. Conjunctival Irritation of Surfactants, Cosurfactants, and Oils by the HET-CAM Assay

Freshly collected fertilized hen eggs (white horn) were incubated for three days at (37 ± 0.5 °C) and at a relative humidity of (40 ± 4%). During incubation, the eggs were kept horizontal inside the trays and were rotated daily to give the embryo a good position and prevent its attachment to one side of the egg. On day three, the eggs were sprayed with methylated spirit and their shells were carefully crack-opened and poured into a growing chamber made of a glass beaker with a piece of cellophane membrane (Glad Wrap) on it. After that, the eggs were examined for embryo viability with an intact CAM and yolk. Eggs with dead embryos, or broken yolk sacs were discarded, others were further incubated [38]. On day 10, a specified amount (200 µL) of each surfactant, cosurfactant, and oil was placed on the CAM and tested for any sign of irritation. NaOH (0.1 M) and normal saline were used as positive (strong irritant) and negative (non-irritant) controls respectively. After time intervals of 0.5, 2, and 5 min, the blood vessels and capillaries were examined for any irritant effects like hyperemia, hemorrhage, and coagulation. A time-dependent numerical score (Table 1) was used to give a single value (cumulative score) was obtained after the summation of the three irritant response scores. This value was interpreted as the irritation potential of the test substance (Table 2).

### 2.3. Statistical Analysis

Data was represented as a mean value minus/plus standard deviation (±SD) of three independent experiments. Student’s tests and one-way analysis of variance (ANOVA) were used for a statistical significance calculation using the software GraphPad Prism (GraphPad Prism software, Inc., San Diego, CA, USA). *p* values < 0.05 were considered as significant.

## 3. Results and Discussion

### 3.1. Identification and Selection of Potential Surfactants and Cosurfactants Based on HLB Calculations

The HLB system provides a systematic approach for selecting mixtures of surfactants and cosurfactants for NE preparation; matching HLB values of surfactants will promote the formation of a physically stable NE [26]. Low values of HLB indicate lipophilicity or less water solubility, while higher values indicate more water-solubility or hydrophilicity [20]. Many researchers have used HLB values during the early stages of formulation development to aid in the selection of appropriate surfactants for a particular purpose [24,40].

The HLB value takes into account the contribution of hydrophilic and lipophilic parts of the surfactant molecule [34]. The type of NE formed could be predicted from the HLB values of surfactants and cosurfactants used in their formulation. HLB values from (3–6) are more likely to produce water in oil (*w*/*o*) NEs, while oil in water (*o*/*w*) type is more likely to be formed in the range of (8–18). The HLB value for all surfactants and cosurfactants of interest in this study was determined according to Davies’ method and compared to literature-reported values (Table 3).

### 3.2. Determination of Physicochemical Properties and Molecular Descriptors of Selected Cosurfactants

The physicochemical properties of cosurfactants can appreciably affect their performance and ability to promote NE formation, stabilization, and performance as drug delivery systems [43]. The calculated physicochemical properties of selected cosurfactants would affect their ability to promote NE formation of a particular type (*o*/*w* or *w*/*o*). As outlined before, cosurfactants exert a dual effect, interfacial and bulk effects. The values of the selected molecular descriptors are shown in Table 4. Log *P* is a measure of the lipophilicity of the unionized part of the cosurfactant in a specific pH range at a particular temperature [44]. Increasing the percentage of carbon (C%) in the backbone of the cosurfactant was associated with higher values of log *P*, lower dielectric constant (DEC), and larger molecular weight (Mwt). The molecular descriptors studied for these cosurfactants are critical to inform their choice for future phase-behavior studies in addition to their impact on the solubility of the hydrophilic drug of interest KT.

### 3.3. Saturation Solubility Studies of Ketorolac Tromethamine in Surfactants, Cosurfactants, and Oils

For developing a successful and stable NE, the saturation solubility of the drug in all excipients is one of the most important criteria [45]. The saturation solubility of KT was determined in all excipients of interest (Table 5). Of the investigated surfactants Tween 60 achieved the highest KT solubility (9.89 ± 0.17 mg/mL), followed by Cremophor RH 40 (9.00 ± 0.21 mg/mL), Tween 20 (7.30 ± 0.20 mg/mL), and Labrasol ALF (6.64 ± 0.13 mg/mL). Whilst one would expect a correlation between HLB of surfactant and KT solubility, this was not the case. However, the difference in KT solubility is significant (*p* < 0.05) amongst all surfactants. Nevertheless, it is worthwhile noting that, the average solubility of KT in high HLB surfactant (HLB >13) is around 6.78 mg/mL whereas for the low HLB surfactant (HLB < 9) it is around 1.96 mg/mL. The differences in HLB between Tween 20, 60, and 80 are insignificant (*p* > 0.05). However, KT solubility difference are significant (*p* < 0.05). One possible explanation for the unusual behavior of Tween 80 could be due to the nature of the fatty acid residue being unsaturated whereas both Tween 60 and 20 are based on saturated fatty acids [22]. The difference in HLB value between the Cremophor EL and Cremophor RH40 is insignificant, yet the difference in KT solubility is. This observation is similar to what was observed with Tweens. One possible explanation could be due to the difference in the molecular structure where Cremophor EL has unsaturation and less polyethylene oxide leading to a higher packing parameters. In both molecules, the unsaturation resulted in less solubility of KT, this could be related to the effect of chain unsaturation on packing parameters, where less intermolecular contact is likely [46]. Other molecular descriptors such as log *P*, DEC are also known to impact ionization potential and water/oil partitioning, respectively. Both could impact solubility of KT in a particular cosurfactant.

Amongst the investigated cosurfactants, Ethylene glycol resulted in the highest solubility for KT (36.84 ± 0.40 mg/mL), followed by propylene glycol (26.23 ± 0.82 mg/mL), butylene glycol (21.09 ± 1.22), pentylene glycol (16.04 ± 0.95), and hexylene glycol (10.36 ± 0.85). Butylene glycol (1,2-butanediol) and its isomer 2,3-butylene glycol (2,3-butanediol) had same HLB value and close physicochemical properties; yet a slight difference between their KT solubility (which was not significant (*p* > 0.05). On the other hand, 2-butyl-2-ethyl-1,3-propandiol, and dimethyl isosorbide had significantly different (*p* < 0.05) HLB values (6.52 and 8.4, respectively) as well as significant difference (*p* < 0.05) in KT solubility. Such difference could be related to the more drastic variation in molecular shape and physicochemical properties that could impact solubility [47]. Furthermore, dimethyl isosorbide has more steric hindrance than 2-butyl-2-ethyl-1,3-propandiol, this could potentially lead to a decrease in the solubility of KT [48].

Transcutol P promoted a relatively high solubility (13.32 ± 0.11 mg/mL) of the drug. To avoid any potential precipitation and phase separation in NE formulations, maximum solubility of the drug is needed inside the oil [45]. On the other hand, reduced solubility of a highly water-soluble drug (like KT) could serve the purpose of delaying drug release, hence providing an opportunity to modulate and prolong the release. Of the investigated oils, Triacetin showed the highest solubility for KT (0.06 ± 0.001 mg/mL), which could be related to the existence of three ester groups that impart some hydrophilic nature to the molecule, followed by ethyl oleate (0.04 ± 0.01 mg/mL), which is a long chain (C20) unsaturated fatty acid ester. Also, no significant difference (*p* > 0.05) was seen when the saturation solubility of the drug was tested in simulated tear fluid (STF) and deionized water which was the highest relative of all excipients.

#### Effect of Cosurfactants Carbon Chain Length, DEC, log P, and HLB Value on the Solubility of Ketorolac Tromethamine

**Figure 2:**
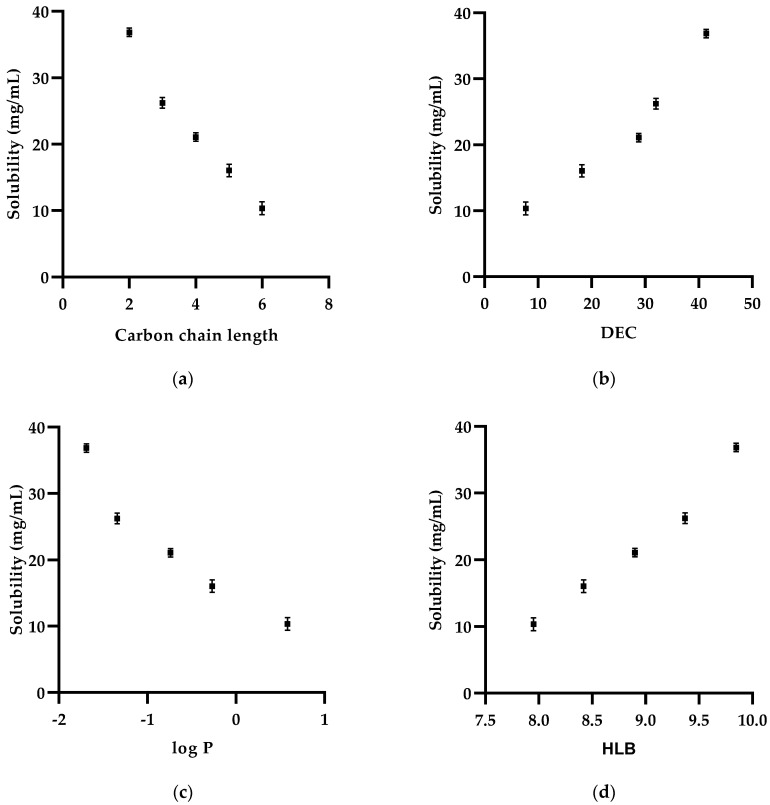
The dependency of KT solubility on (**a**) carbon chain length, (**b**) dielectric constant (**c**) log *P,* and (**d**) HLB of the diol cosurfactant (ethylene, propylene, butylene, pentylene, and hexylene glycols). Results are expressed as the mean ± SD, *n* = 3.

The solubility of KT decreased significantly (*p* < 0.05) as the number of backbone carbon atoms increased from 2 to 6 (Figure 2a); this could be related to the physicochemical properties of the drug and the cosurfactants used. A relationship has been established between solubility and DEC. DEC is an indirect measure of the ionization potential of the molecule as well as the tendency of a particular solvent to promote such ionization [49]. Changes in dielectric constant have a great effect on the solubility of electrolytes (like KT) in particular; higher DEC causes more ionization of KT, hence more solubility [50]. Increasing DEC of cosurfactants caused a significant (*p* < 0.05) increase in solubility of KT (Figure 2b). log *P* is regarded as an essential parameter for predicting solubility [51]. Many studies demonstrate increasing solubility of drugs with decreasing lipophilicity (log *P*) [51,52]. The dependence of the solubility of KT on log *P* of cosurfactants is shown in Figure 2c. Furthermore, the HLB value could be used as an indication of the solubility behavior, lower HLB values indicate higher lipophilicity while higher values indicate more hydrophilicity, hence more solubility [26]. From the results, it was seen that as the cosurfactants carbon atom numbers (carbon chain length) increased from two atoms (ethylene glycol) to six atoms (hexylene glycol), the HLB value decreased significantly (*p* < 0.05) (Figure 2d). A previous study reports that with 1,2-alkandiol molecules increasing carbon chain length imparts more lipophilicity to the molecule thus increasing carbon chain length results in a decrease in HLB value [22]. The investigated alkanediols are the only group of cosurfactant molecules that fulfilled the definition of “homologous series” where there is a small and incremental change in molecular structure (one carbon atom at a time). The position of the two hydroxyl groups remained constant (1,2 diols). This gradual and systematic change in molecular structure allowed clear and unambiguous correlation with molecular descriptors and KT solubility as well as molecular structure. On the other hand, none of the other surfactants, cosurfactants, and oils investigated in this study had such a close molecular structure similarity. Generally, in this study the excipients with higher HLB values (hydrophilic) were able to dissolve KT to a greater extent than those with lower HLB values (Table 3 and Table 5), which is due to the hydrophilic nature of the drug (aqueous solubility is >200 mg/mL, Pka = 3.5, log *P* = 2.28).

### 3.4. Conjunctival Irritation of Surfactants, Cosurfactants, and Oils Determined by the HET-CAM Assay

**Figure 3:**
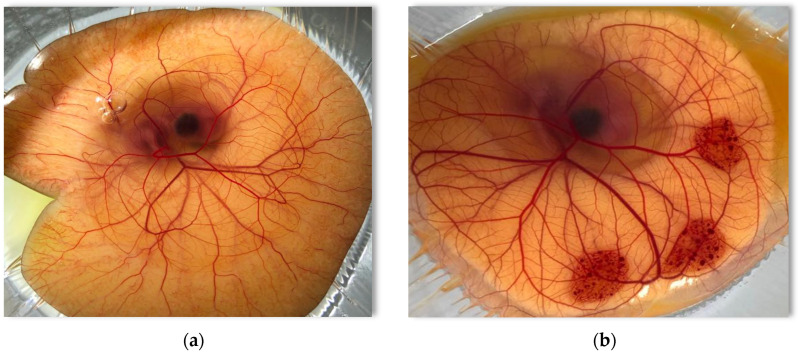
Vascular responses to control substances applied to the CAM surface 5 min post-application. (**a**) Normal saline (negative control), (**b**): 0.1 M NaOH (positive control).

Ocular irritation is likely to be an issue in NE formulation development, due to their relatively high surfactant and cosurfactant content. Ocular irritation is a source of patient discomfort where it could lead to discontinuation of the medication [53]. The HET-CAM assay has been used for the evaluation of conjunctival responses to test substances. The chorioallantois is a well-accepted ex vivo model of the human conjunctiva [32,54]. The vascular and inflammatory responses of CAM to injury are immediate. Those responses are similar to those produced by the human conjunctival tissue [55]. The vascular response of the CAM surface to the negative control substance (normal saline) and positive control substance (NaOH) are shown in Figure 2. Normal saline showed no effect on the surface of the CAM (Figure 3a) while NaOH produced a very strong irritation response with complete blood vessel lysis, clotting, and coagulation (Figure 3b).

Table 6 summarizes the conjunctival irritation potential results of all the surfactants, cosurfactants, and oils of interest as determined using the HET-CAM assay. Tweens and spans were shown to be practically non-irritant. It has been previously reported that formulations containing Spans possessed good ocular tolerability with a non-irritant effect [22]. Cremophor EL, Cremophor RH 40, Labrafills, Triacetin, and Labrafac lipophile WL 1349 were also deemed to be non-irritant. Cremophor EL has been previously reported to be safe to the eye in concentrations up to 30% (*w*/*w*) [56]. Labrasol ALF has been reported to be non-irritant at concentrations of 0.5–3% (*v*/*v*) and slightly irritant at higher concentrations [57].

1,2-alkanediols with 2–3 carbon chain length were reported to be only slightly irritant, 1,2-butanediol was a moderate irritant, while carbon chain length (5–8) were strong irritants [58]. In this study, propylene glycol showed slight lysis of blood vessels and thus a slight irritant effect when used alone, butylene glycol was a moderate irritant, pentylene glycol and hexylene glycol showed a strong irritant effect and caused severe damage of blood vessels. It has been previously reported that undiluted propylene glycol solutions may cause weak conjunctival redness, while diluted solutions were non-irritant [59]. The gradual and systematic change in molecular structure of 1,2 alkanediol (homologous series) allowed clear and unambiguous correlation between molecular structure and ocular irritation. Transcutol P was deemed to be slightly irritant, this result is in agreement with those obtained by Butt et al. [55] and Liu et al. [60]. Triacetin and Labrafac lipophile WL 1349 were practically non-irritant, while ethyl oleate, Labrafac PG, and isopropyl myristate were slight irritant.

## 4. Conclusions

A range of nonionic surfactants, cosurfactants and oils were screened for the suitability as NE excipients for ocular administration of KT. The solubility of KT was found to be affected by the physicochemical properties of surfactants and cosurfactants (carbon chain length, HLB value, dielectric constant, and log *P*). Only homologous series of alkanediols (C2–C6) showed a clear correlation between calculated molecular descriptors and KT solubility. This systematic change in molecular structure allowed clear and unambiguous correlation of molecular descriptors and KT solubility as well as molecular structure and ocular irritation. On the other hand, none of the other surfactants, cosurfactant and oils investigated in this study had such a close molecular structure similarity, hence the lack of correlation between molecular descriptors and KT solubility as well as molecular structure and ocular irritation. All the investigated nonionic surfactants were deemed to be practically non-irritant except for Labrasol ALF, which was found to be slightly irritant. All cosurfactants investigated were found to be irritant, albeit to varying extents. Of the investigated excipient and considering both KT solubility and ocular irritation results; Tween 60, Cremophor RH 40 and Tween 20 would be the surfactants to be taken forward to use in developing NE formulation for ocular KT delivery. As for studied cosurfactants, ethylene glycol and propylene glycol would be the candidates of choice, albeit being slightly irritant. Triacetin and Labrafac lipophile WL 1349 would be the oils of choice.

## Figures and Tables

**Table 1:** Time-dependent numerical scores for each of the three irritant responses [39].

Effect	Score		
Time (min)	0.5	2.0	5.0
Hyperemia	5	3	1
Hemorrhage	7	5	3
Clotting or Coagulation	9	7	5

**Table 2:** Irritation potential interpretation of various cumulative scores [39].

Cumulative Score	Irritation Potential
0.0–0.9	Practically none
1.0–4.9	Slight
5.0–8.9	Moderate
9.0–21.0	Strong

**Table 3:** Selected surfactants and cosurfactants with their calculated and reported HLB values.

Surfactants & Cosurfactants	Calculated HLB	Reported HLB
Tween 20	16.67	16.70 [34]
Tween 60	13.82	14.90 [34]
Tween 80	13.82	15.0 [34]
Span 20	8.67	8.60 [34]
Span 80	5.82	4.30 [34]
Cremophor RH 40	15.65	15.0 [41]
Cremophor EL	14.0	13.0 [41]
Labrasol ALF	14.69	14.0 [40]
Labrafil M2125 CS	5.20	4.0 [40]
Labrafil M1944 CS	5.20	4.0 [40]
Labrafil M2130 CS	5.20	4.0 [40]
Ethylene glycol	9.85	9.85 [42]
Propylene glycol	9.37	9.38 [42]
Butylene glycol	8.90	8.90 [42]
Pentylene glycol	8.42	8.43 [19]
Hexylene glycol	7.95	7.95 [19]
Dimethyl isosorbide	8.40	-
2-Butyl-2-ethyl-1,3-propanediol	6.52	-
2,3-Butanediol	8.90	8.90 [42]
Transcutol P	8.61	4.20 [40]

**Table 4:** Physicochemical properties (molecular descriptors) of selected cosurfactants (diols).

Cosurfactants (Diols)	Chemical Formula	C No ^1^	log *P* ^1^	DEC ^1^	Mwt ^1^ (g/mol)	C% ^1^
Ethylene glycol (1,2-ethanediol)	C2H6O2	2	−1.69	41.4	62.07	38.70
Propylene glycol (1,2-propanediol)	C3H8O2	3	−1.34	32.0	76.10	47.35
Butylene glycol (1,2-butanediol)	C4H10O2	4	−0.74	28.8	90.12	53.30
Pentylene glycol (1,2-pentanediol)	C5H12O2	5	−0.27	18.2	104.15	57.66
Hexylene glycol (1,2-hexanediol)	C6H14O2	6	0.58	7.70	118.17	60.98

^1^ C No; carbon atom number, log *P*; logarithm of partition coefficient in octanol/water, DEC; dielectric constant, Mwt; molecular weight, C%; carbon content weight percent.

**Table 5:** Saturation solubility of KT in different excipients (surfactants, cosurfactants, oils, deionized water, and simulated tear fluid).

Excipients	Solubility ^1^ (mg/mL)	Excipients	Solubility ^1^ (mg/mL)
Tween 20	7.30 ± 0.20	Pentylene glycol	16.04 ± 0.95
Tween 60	9.89 ± 0.17	Hexylene glycol	10.36 ± 0.85
Tween 80	3.82 ± 0.41	Dimethyl isosorbide	4.54 ± 0.55
Span 20	5.01 ± 0.27	2-butyl-2-ethyl-1,3-propandiol	9.55 ± 0.60
Span 80	3.18 ± 0.37	2,3-butanediol	22.93 ± 0.24
Cremophor RH 40	9.00 ± 0.21	Transcutol P	13.32 ± 0.11
Cremophor EL	4.03 ± 0.38	Triacetin	0.06 ± 0.001
Labrasol ALF	6.64 ± 0.13	Ethyl Oleate	0.04 ± 0.01
Labrafil M2125 CS	0.48 ± 0.06	Labrafac PG	0.01 ± 0.002
Labrafil M1944 CS	0.33 ± 0.03	Labrafac lipophile WL 1349	0.01 ± 0.01
Labrafil M2130 CS	0.80 ± 0.04	Isopropyl Myristate	0.01 ±0.02
Ethylene glycol	36.84 ± 0.40	Simulated tear fluid	199.2 ± 7.40
Propylene glycol	26.23 ± 0.82	Deionized water	217.1 ± 1.13
Butylene glycol	21.09 ± 1.22		

^1^ Mean ± SD, *n* = 3.

**Table 6:** Ocular irritation potential calculated from the cumulative scores of the vascular responses of the CAM.

Surfactants, Cosurfactants & Oils	Irritation Potential (Numerical Score)			
	Practically Non (0.0–0.9)	Slight (1.0–4.9)	Moderate (5.0–8.9)	Strong (9.0–21.0)
KT solution		√		
Tween 20	√			
Tween 60	√			
Tween 80	√			
Span 20	√			
Span 80	√			
Cremophor RH 40	√			
Cremophor EL	√			
Labrasol ALF		√		
Labrafil M2125 CS	√			
Labrafil M1944 CS	√			
Labrafil M2130 CS	√			
Ethylene glycol		√		
Propylene glycol		√		
Butylene glycol			√	
Pentylene glycol				√
Hexylene glycol				√
Dimethyl isosorbide		√		
2-Butyl-2-ethyl-1,3-propandiol			√	
2,3-Butanediol			√	
Transcutol P		√		
Triacetin	√			
Ethyl Oleate		√		
Labrafac PG		√		
Labrafac lipophile WL 1349	√			
Isopropyl Myristate		√		
Normal saline	√			
NaOH (0.1 M)				√

## Data Availability

Not applicable.
